# Nuclear Factor I/A Controls A-fiber Nociceptor Development

**DOI:** 10.1007/s12264-020-00486-7

**Published:** 2020-03-28

**Authors:** Lu Qi, Guangjuan Yin, Yongchao Zhang, Yeqi Tao, Xiaohua Wu, Richard M. Gronostajski, Mengsheng Qiu, Yang Liu

**Affiliations:** 1 College of Life Sciences, Zhejiang University, Hangzhou, 310058 China; 2 Zhejiang Key Laboratory of Organ Development and Regeneration, Institute of Life Sciences, Hangzhou Normal University, Hangzhou, 310036 China; 3 Department of Biochemistry, Program in Genetics, Genomics and Bioinformatics, Center of Excellence in Bioinformatics and Life Sciences, State University of New York at Buffalo, Buffalo, NY 14203 USA

**Keywords:** Dorsal root ganglion, Acute pain, Pinprick pain, Npy2r, Nppb, Nociceptor, A-fiber mechanonociceptor

## Abstract

Noxious mechanical information is transmitted through molecularly distinct nociceptors, with pinprick-evoked sharp sensitivity *via* A-fiber nociceptors marked by developmental expression of the neuropeptide Y receptor 2 (Npy2r) and von Frey filament-evoked punctate pressure information *via* unmyelinated C fiber nociceptors marked by MrgprD. However, the molecular programs controlling their development are only beginning to be understood. Here we demonstrate that Npy2r-expressing sensory neurons are in fact divided into two groups, based on transient or persistent Npy2r expression. Npy2r-transient neurons are myelinated, likely including A-fiber nociceptors, whereas Npy2r-persistent ones belong to unmyelinated pruriceptors that co-express Nppb. We then showed that the transcription factors NFIA and Runx1 are necessary for the development of Npy2r-transient A-fiber nociceptors and MrgprD^+^ C-fiber nociceptors, respectively. Behaviorally, mice with conditional knockout of *Nfia*, but not *Runx1* showed a marked attenuation of pinprick-evoked nocifensive responses. Our studies therefore identify a transcription factor controlling the development of myelinated nociceptors.

## Introduction

The past decade has seen the characterization of a cohort of molecularly distinct somatosensory neurons transmitting noxious or innocuous mechanical information, three of which were addressed in this study. Firstly, a large group of unmyelinated mechanical nociceptors in dorsal root ganglia (DRG), which are marked with the expression of the mas-related G protein coupled receptor (MrgprD), exclusively innervate the skin epidermis and transmit light punctate pressure information [1–3]. Secondly, another group of unmyelinated mechanoreceptors respond to gentle stroking across the skin, and these neurons are marked by the expression of the vesicular glutamate transporter 3 (Vglut3), tyrosine hydroxylase (TH), and the cytokine TAFA4 [4–7]. Notably, although TH^+^ neurons are called C-fiber low threshold mechanoreceptors (C-LTMRs), they can nonetheless positively and negatively modulate the transmission of noxious mechanical information [7]. Thirdly, in humans pinprick-evoked sharp mechanical pain percepts are mediated *via* myelinated nociceptors, referred here to as A-fiber nociceptors, and in mice they can be genetically marked by *Npy2r-Cre* [8]. In *Npy2r-Cre* transgenic mice, the Cre DNA recombinase is driven from the promoter of the gene encoding the neuropeptide Y receptor 2 (Npy2r) [8]. Npy2r-Cre-marked DRG neurons include a subset of nociceptors that express the nerve growth factor receptor TrkA and the calcitonin gene-related peptide (CGRP), and ablation of these neurons leads to marked attenuation of pinprick-evoked mechanical sensitivity [8].

To date, the genetic programs controlling the development of different classes of mechanical nociceptors are still not fully understood. All nociceptors and unmyelinated sensory neurons are derived from sensory neurons whose embryonic survival is dependent on the nerve growth factor receptor TrkA [9, 10]. TrkA lineage neurons are formed in two waves in thoracic and lumbar DRGs, the early one forming A-fiber nociceptors dependent on the proneural gene *Neurog2*, and the later one forming unmyelinated neurons dependent on the proneural gene *Neurog1* [9–12]. A number of studies then showed that the runt domain transcription factor Runx1 is mainly associated with the Neurog1-dependent later wave of sensory neurons, and Runx1 is necessary for the specification of MrgprD^+^ nociceptors [6, 13, 14], and both Runx1 and the transcription factor Zfp521 are required for establishing the molecular features of TH/Vglut3^+^ C-LTMRs [15]. However, the transcription factor controlling the development of A-fiber nociceptors remains unknown. In this study, we set out to reveal separate genetic programs controlling the development of myelinated nociceptors *versus* unmyelinated nociceptors/LTMRs.

## Materials and Methods

### Animals

The generation of mice carrying the floxed *Runx1* allele (RRID:MGI:4358522), *Wnt1-Cre* and *Nav1.8-Cre* transgenic mice has been described previously [16–18]. *Nfia*^*F/F*^ mice were provided by Dr. Richard M. Gronostajski [19]. The morning that vaginal plugs were observed was considered E0.5. For immunohistochemical studies, we used mice at E11.5–E16.5 and P0–P30. For behavioral analyses, 6–10 week-old mutant and control littermates of either sex were used, and behavioral measurements were conducted with genotypes blinded for the investigators. All behavioral test protocols were approved by the Institutional Animal Care and Use Committee at Hangzhou Normal University.

### *In Situ* Hybridization (ISH) and Immunofluorescence (IF)

Mice were anesthetized with CO_2_ and perfused with 4% paraformaldehyde in phosphate buffered saline (pH 7.4) at 4 °C. The spinal cord and DRG were dissected and post-fixed at 4 °C overnight. Tissues were cryo-protected in 25% sucrose, embedded with O.C.T., and stored at − 80 °C before use. The procedures for ISH and probe preparation have been described previously [13]. The primer information for preparing all probes was from http://www.brain-map.org. The primary antibodies used in IF staining were mouse anti-NF200 monoclonal antibody (Abcam Cat# ab82259, RRID:AB_1658500, 1:500), rabbit anti-c-Fos (Millipore Cat# ABE457 RRID:AB_2631318, 1:1000), rabbit anti-NF1A (Active Motif Cat# 39036, RRID:AB_2335600), 1:1000), and mouse anti-CGRP (Abcam Cat# ab81887, RRID: AB_1658411, 1:800). FITC-conjugated IB4 was from Sigma-Aldrich (Cat#: L2895). The secondary antibodies were from Life Technologies. The ISH/IF double staining was performed as previously described [14]. We performed double ISH of Nppb mRNA labeling with DIG-AP and Npy2r mRNA labeling with botin-488. After ISH, each section was re-photographed and the *in situ* signals were pseudo-colored and superposed onto the GFP signal with Adobe Photoshop software. Quantitative analysis was determined by analyzing lumbar-level DRGs from at least three groups of mice. Only cells with clearly visible nuclei were counted.

### Behavioral Studies

All animals were acclimatized to the behavioral testing apparatus in 3–5 ‘habituation’ sessions. All acute pain behavioral tests were performed as previously described with minor modifications [8, 20, 21]. In the rotarod test, mice were first trained on the accelerating rotarod. Training sessions consisted of mice being placed on a rotarod moving at 5 rpm for 10 min so that they could stay on the rotarod for the entire 10 min. After 3–5 training sessions, mice were subjected to a full rotarod test, with the rotorod accelerating from 5 rpm to 40 rpm over 5 min. The time to fall was automatically recorded. The rotarod latency was determined as the average of 3 trials per animal performed at 30-min intervals. To measure light touch sensitivity, mice were placed on an elevated wire grid and habituated for 30 min. The plantar surface of the hindpaw was stimulated by light stroking with a paintbrush, from heel to toe. In each test, no evoked movement was scored as 0, and walking movement or brief paw lifting (< 1 s) was scored as 1. For each mouse, the cumulative score from three tests was used as a measure of the touch response. To measure radiant heat pain by the Hargreaves test, mice were placed on glass plate and habituated in plastic chambers. The plantar surface was exposed to 20% beam intensity of radiant light. The latency to paw withdrawal was calculated for 3 trials per animal, with a 10-min interval between trials. The cut-off latency to avoid tissue damage was 20 s. To measure cold pain, mice were placed on a plastic plate (0.5 mm thick) and the place beneath the hindpaw was contacted with dry ice. The latency to hindpaw withdraw was measured. All animals were tested 3 times sequentially with a minimum of 10 min between tests. In von Frey assays, mice were placed on an elevated wire grid and the plantar surface of the hindpaw was stimulated with calibrated von Frey filaments with 2 s of bending duration. The hindpaw withdrawal threshold was determined by Dixon’s up-down method [22]. For the pinprick response, a sharp steel needle (FST, 10130-10) was glued to the tip of a 1.0-g von Frey filament and gently applied to the plantar surface of the hindpaw without penetrating the skin. A scoring system was used according to the extent of the response: 0, no response; 1, quick move, look around to see what happened; 2, brief quick lift or withdrawal or removal of hindpaw; 3, brief quick shakes of hindpaw, or jumps; 4, based on score 3, 1–2 extra high-frequency shakes of hindpaw; 5, based on score 3, 3–4 extra high frequency shakes of hind paw; and 6, based on score 3, 5–7 extra high frequency shakes of hind paw. In the pinch test, an alligator clip was applied to the ventral skin surface between the footpad and the heel of the hindpaw. The cut-off threshold was set at 60 s to avoid tissue damage. Each mouse was confined in a plexiglas chamber, with video recording from below to calculate the licking duration [23]. Both males and females were used. Investigators performed these behavioral analyses blinded to genotypes.

### c-Fos Induced by Pinprick

Two-month-old *Nfia*^*F/F*^*;Wnt1-Cre, Runx1*^*F/F*^*;Wnt1-Cre* mice and their littermates were anesthetized with 2% isoflurane. A sharp steel needle glued to the tip of a 1.0-g von Frey filament was poked into the left planta ~1200 times in a 20-min period (frequency, 1 Hz). One and a half hours later, the L4–L5 spinal segments were dissected and treated as previously described [24]. Frozen spinal cord sections (14 µm) were immuno-stained for c-Fos according to the manufacture’s instructions.

### Cell Counting and Statistical Analysis

The L4 or L5 lumbar DRG was dissected from at least three pairs of mutant and control mice. Three to four mutant or control DRGs were used to prepare eight sets of adjacent sections at 12 µm thickness. Only cells containing nuclei and showing levels of expression or staining clearly above background were counted. Representative data are from experiments that were replicated biologically at least three times with similar results. All data are presented as the mean and its standard error (mean ± SEM), and the difference between control and mutant samples was subjected to a two-tailed, unpaired Student’s *t* test. Differences among multiple groups were analyzed using one-way ANOVA followed by the Bonferroni *post-hoc* analysis. Differences with *P* < 0.05 were considered statistically significant.

## Results

### Dynamic Expression of Npy2r Marks Two Groups of DRG Neurons

**Fig. 1:**
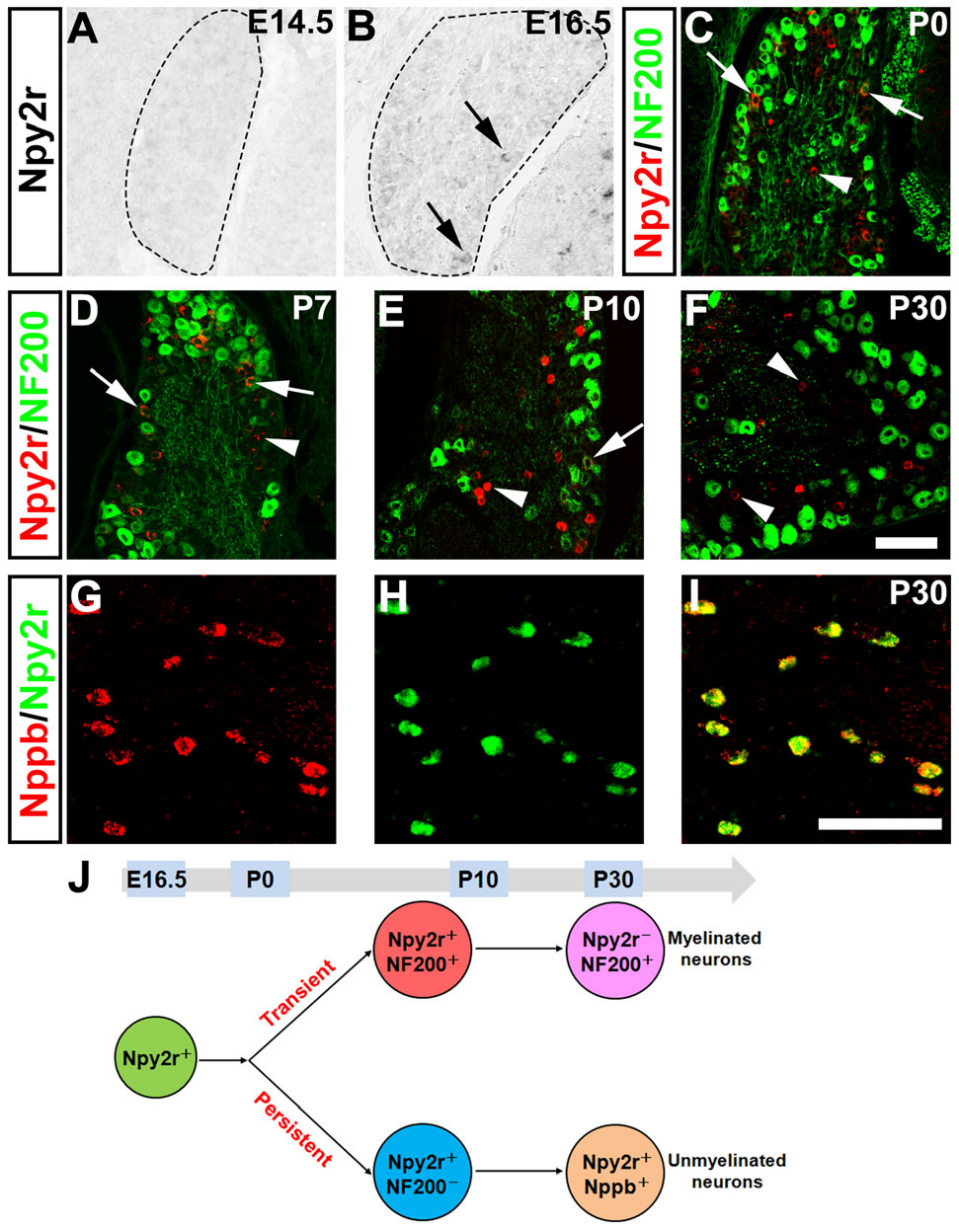
Dynamic Npy2r expression in developing DRGs. **A**–**F***In situ* hybridization (ISH) with the Npy2r probe in sections through lumbar DRGs from wild-type mice at E14.5 (**A**), E16.5 (**B**), P0 (**C**), P7 (**D**), P10 (**E**) and P30 (**F**) developmental stages (P0–P30 sections co-stained with NF200; arrowheads, Npy2r^+^;NF200^-^ neurons; arrows, Npy2r^+^;NF200^+^ neurons). **G**–**I** Double stained ISH showing that Nppb and Npy2r are completely co-labeled. **J** Schematic of dynamic Npy2r expression that reveals two subsets of Npy2r lineage neurons. Scale bars, 100 µm.

We first set out to clarify the expression of Npy2r in the developing DRG, due to conflicting results from different approaches. One study showed that DRG neurons labeled by green fluorescent protein (GFP) in transgenic *Npy2r*^*GFP*^ mice are Aβ-fiber rapid-adapting low threshold mechanoreceptors or RA-LTMRs [5]. In another study, the Npy2r lineage neurons marked by transgenic *Npy2r-Cre* were shown to be peptidergic A-fiber nociceptors that are required to sense pinprick-evoked mechanical stimuli [8]. However, single-cell RNA-seq studies revealed that high-level Npy2r expression is restricted to unmyelinated pruriceptors marked by expression of the natriuretic peptide type B or Nppb [25–27]. With these controversies, we therefore decided to examine Npy2r expression in developing DRGs by performing ISH on DRG sections from different stages (from E14.5 to P30). We found that the Npy2r expression was initiated at E16.5 in lumbar DRGs (Fig. 1A, B), and that expression was still detected at the young adult stage (P30) (Fig. 1F). To assess the identity of these Npy2r-expressing neurons, we performed double staining that combined Npy2r ISH with immunostaining against the neurofilament protein NF200, which is a marker for myelinated neurons. We found that a subpopulation of Npy2r-expressing neurons were myelinated (40.6% ± 3.2% of all Npy2r^+^ neurons) at P0 (Fig. 1C), as indicated by co-expression with NF200. This co-staining was reduced to 31.6% ± 3.2% at P7, 16.4% ± 1.3% at P10, and virtually none (1/189) at the young adult stage (P30) (Fig. 1D–F). Thus, Npy2r-expressing neurons comprise at least two groups: (1) the NF200^+^ myelinated group expressing Npy2r transiently (or the expression is downregulated to levels not detected by ISH at P30), and (2) the NF200-negative unmyelinated group persistently expressing Npy2r. Consistent with single-cell RNA-seq data [25–27], we further found that all Npy2r-persistent neurons co-expressed Nppb in P30 DRGs (Fig. 1G–I). It should be noted that DRG neurons marked by *Npy2r-Cre*, which have been reported to be A-fiber mechanoreceptors [8], most likely represent the Npy2r-transient population characterized here, and in that study, those investigators did not determine if *Npy2r-Cre* actually also marks unmyelinated Npy2r-persistent Nppb^+^ pruriceptors [28, 29]. Regardless, our results suggested that the Npy2r lineage DRG neurons include Npy2r-transient myelinated A fiber sensory neurons, plus Npy2r-persistent unmyelinated pruriceptors (Fig. 1J). Next, we investigated the roles of two transcription factors, nuclear factor I/A (NFIA) and Runx1, in controlling the development of these Npy2r-lineage DRG neurons.

### Detection of NFIA Expression in Npy2r-transient Myelinated Neurons

**Fig. 2:**
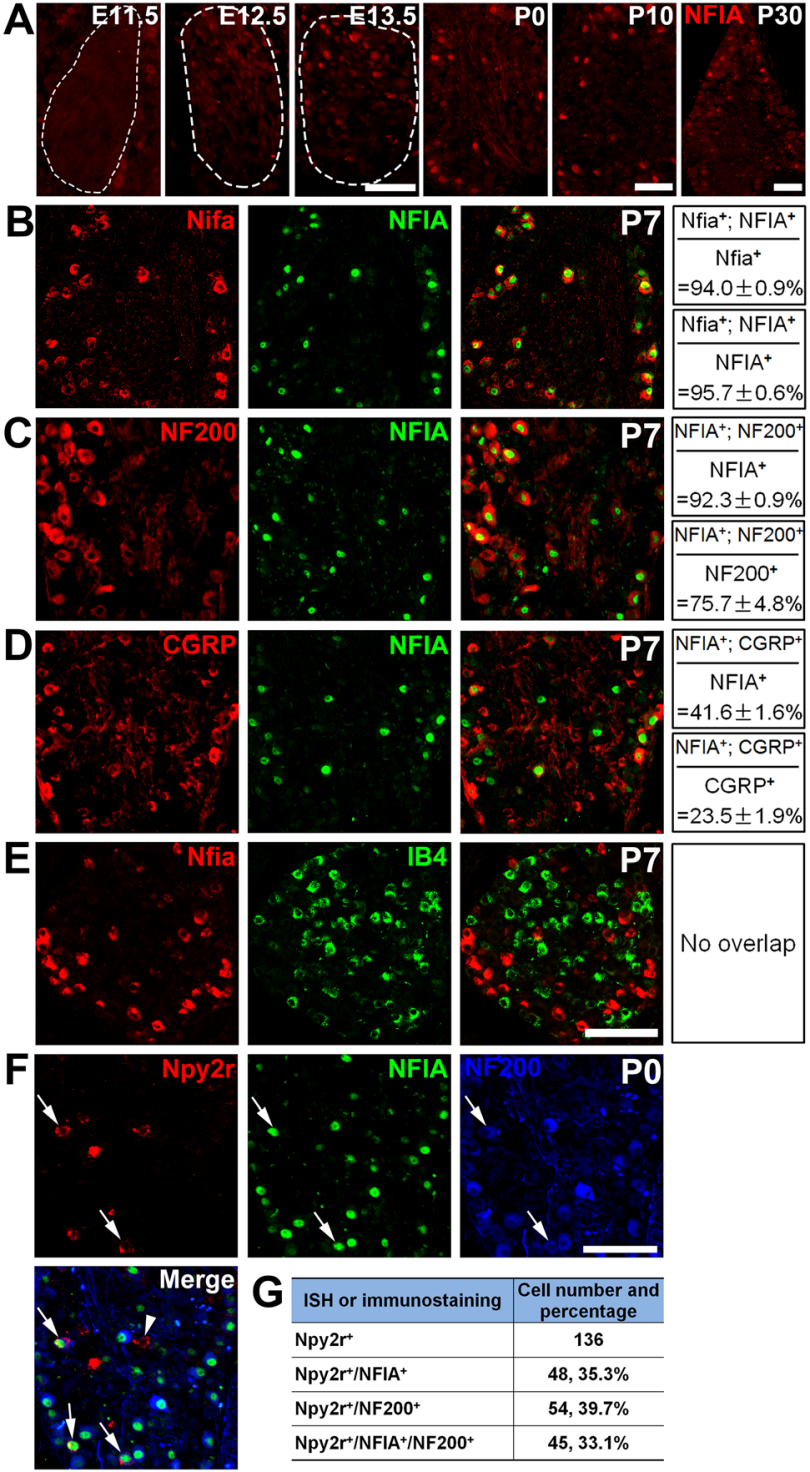
NFIA expression in developing DRG neurons and its association with Npy2r-transient neurons. **A** Representative images of anti-NFIA immunostaining showing its expression in a subset of DRG neurons at indicated stages. **B** Images of *Nfia* mRNA revealed by ISH combined with NFIA immunostaining (green) in DRG sections from P7 wild-type mice. **C, D** Double staining of NFIA and indicated molecular markers in DRG sections from P7 wild-type mice. **E** Double staining of *Nfia* mRNA by ISH plus IB4 labeling. **F** Images showing *Npy2r* mRNA by ISH combined with immunostaining against NFIA (green) and NF200 (blue) in DRG sections from P0 wild-type mice (arrowhead, an Npy2r^+^;NF200^-^ neuron; arrows, Npy2r^+^ cells co-expressing NFIA and NF200). **G** Tabular representation of various neuronal populations (18 sections from 3 mice). Scale bars, 100 µm.

**Fig. 3:**
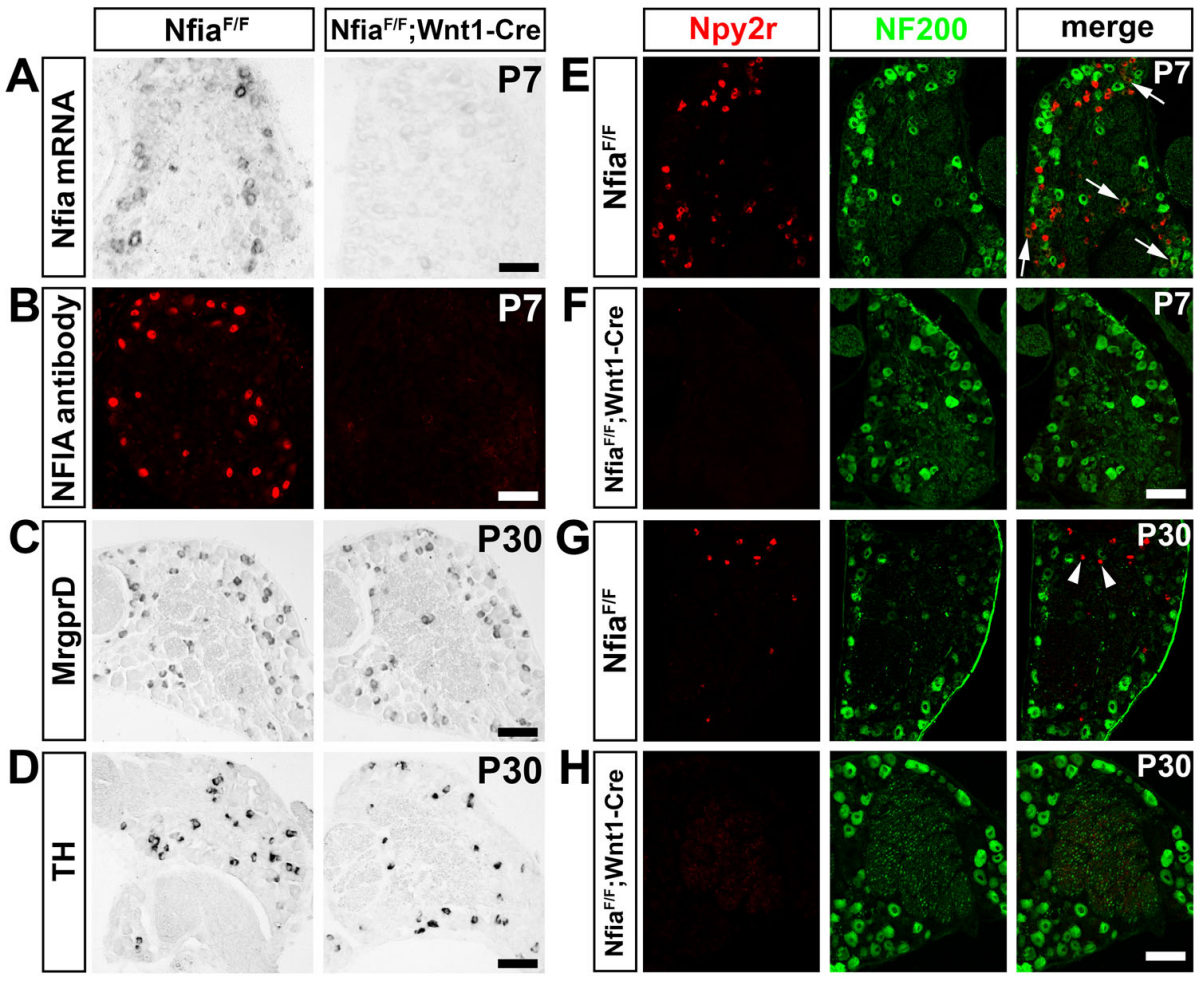
Selective impairment of Npy2r neuron development in *Nfia*^*F/F*^*;Wnt1-Cre* mutants. **A, B** Nfia knockout confirmed by loss of *Nfia* mRNA by ISH (**A**) and NFIA protein by immunostaining (**B**). **C** The numbers of MrgprD^+^ neurons in *Nfia*^*F/F*^*;Wnt1-Cre* mutants (36.0% ± 1.1%, *n* = 7) were comparable to those in control mice (35.2% ± 1.0%, *n* = 6) (Student’s unpaired *t-*test, *P* = 0.6064, *t*_(11)_ = 0.5303). **D** The numbers of TH^+^ neuron in *Nfia*^*F/F*^*;Wnt1-Cre* mutants (11.0% ± 0.4%) were comparable to those in control mice (10.4% ± 0.4%) (*n* = 6/group; Student’s unpaired *t-*test, *P* = 0.3516, *t*_(10)_ = 0.9769). **E**–**H** Complete loss of Npy2r expression in P7 and P30 *Nfia*^*F/F*^*;Wnt1-Cre* mutant mice (arrows, Npy2r^+^;NF200^+^ neurons; arrowheads, persistent Npy2r^+^;NF200^-^ neurons). Scale bars, 100 µm.

Using immunostaining, we discovered that NFIA is expressed in a subset of DRG neurons (Fig. 2A). Expression was first detected at ~E12.5–E13.5 and persisted to young adult ages (Fig. 2A). The fidelity of this NFIA antibody was indicated by the high co-labelling rate of the immunostaining signal and the *Nfia* mRNA in P7 DRGs (Fig. 2B), as well as by the loss of immunostaining signals in mice with conditional knockout (see below, Fig. 3B). Double staining showed that by P7, NFIA was mainly expressed in myelinated neurons, with 92.3% ± 0.9% of NFIA^+^ cells co-expressing NF200 (Fig. 2C), and NFIA^+^ cells accounting for 75.7% ± 4.8% of NF200^+^ cells. Previous studies have shown that NPY2R-lineage A-fiber nociceptors co-express CGRP [8]. Consistent with this, 41.6% ± 1.6% of NFIA^+^ neurons co-expressed CGRP (Fig. 2D), and 23.5% ± 1.9% of CGRP^+^ neurons co-expressed NFIA. In contrast, *Nfia* mRNA was not detected in a large group of non-peptidergic, unmyelinated neurons marked by co-staining with isolectin B4 (IB4) (Fig. 2E).

With predominant expression of NFIA in NF200^+^ neurons at P7, we next determined whether NFIA is expressed in Npy2r-transient NF200^+^ neuron at neonatal stages, before Npy2r is downregulated in NF200^+^ neurons at young adult stages. We co-stained sections of P0 DRGs, and found that 33.1% of Npy2r^+^/NF200^+^ cells showed detectable NFIA expression (Fig. 2F, G), indicating that NFIA is at least expressed in a subset of Npy2r-transient A-fiber sensory neurons. As described below, NFIA is necessary for Npy2r expression, raising the possibility that the remaining 67% of Npy2r^+^ neurons (including the NF200^+^ and NF200^-^ subsets) might have switched off NFIA expression during embryonic development.

### NFIA is Necessary for Npy2r Lineage Neuron Development

To determine the role of NFIA in controlling sensory neuron development, we generated *Nfia* conditional knockout (CKO) mice by crossing *Nfia*^*F/F*^ mice [19] with *Wnt1-Cre,* in which the Cre recombinase is expressed in the neural crest stem cells that give rise to all peripheral neurons and in spinal cord neurons [16]. In the resulting *Nfia*^*F/F*^*;Wnt1-Cre* mice, NFIA expression in the DRG, detected by ISH and immunostaining, was completely eliminated (Fig. 3A, B). As described above, NFIA is not expressed in non-myelinated IB4^+^ neurons, most of which are MrgprD^+^ polymodal nociceptors [2, 3]. Consistent with this, the percentages of DRG neurons expressing MrgprD were not different between *Nfia*^*F/F*^*; Wnt1-Cre* mice (36.0% ± 1.1%, *n* = 7) and control littermates (35.2% ± 1.0%, *n* = 6; *t-*test, *P* = 0.6064, *t*_(11)_ = 0.5303) (Fig. 3C). The percentage of DRG neurons expressing TH was also unaffected (control: 10.4% ± 0.4%, *n* = 6; CKO: 11.0% ± 0.4%; *n* = 6; *t-*test, *P* = 0.3516, *t*_(10)_ = 0.9769) (Fig. 3D), suggesting that NFIA is dispensable for the development of MrgprD^+^ polymodal nociceptors and TH^+^ C-LTMRs. In marked contrast, the expression of Npy2r was completely eliminated in DRGs of *Nfia*^*F/F*^*;Wnt1-Cre* mutant mice at all stages tested, including P0 (data not shown), P7 (Fig. 3E, F), and P30 (Fig. 3G, H), indicating that NFIA is necessary for the development of the whole lineage of Npy2r neurons, including both the Npy2r-transient myelinated and Npy2r-persistent unmyelinated subsets.

### Pinprick-evoked Mechanical Sensitivity is Markedly Attenuated in *Nfia* Conditional Knockout Mice

**Fig. 4:**
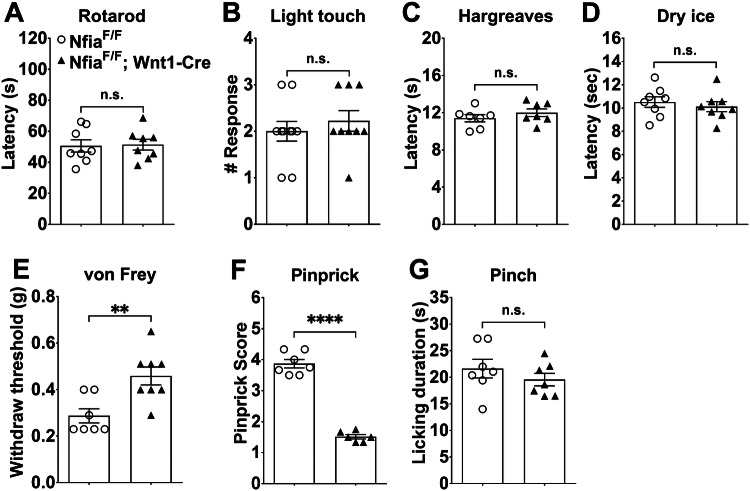
Attenuated pinprick-evoked responses in conditional *Nfia* null mice. **A** General motor coordination, assessed by the accelerating rotarod test, in *Nfia*^*F/F*^*;Wnt1-Cre* and *Nfia*^*F/F*^ control mice (*n* = 8/group, Student’s unpaired *t-*test, *P* = 0.8812, *t*_(14)_ = 0.1521). **B** Normal reflex responses to light touch in the dynamic brush assay in *Nfia*^*F/F*^ control (*n* = 10) and *Nfia*^*F/F*^*;Wnt1-Cre* CKO mice (*n* = 9) (Student’s unpaired *t-*test, *P* = 0.478, *t*_(17)_ = 0.7255). **C** Normal reflex responses to radiant heat in the Hargreaves assay (*n* = 7/group, Student’s unpaired *t-*test, *P* = 0.318, *t*_(12)_ = 1.042). **D** Normal reflex responses to noxious cold in the dry ice assay (*n* = 8/group, Student’s unpaired *t-*test, *P* = 0.5403, *t*_(14)_ = 0.6277). **E** Withdrawal thresholds in response to von Frey filament stimulation in *Nfia*^*F/F*^*;Wnt1-Cre* CKO (*n* = 8) and *Nfia*^*F/F*^ control mice (*n* = 7) (Student’s unpaired *t-*test, *P* = 0.0045, *t*_(13)_ = 3.425). **F** Pinprick scores evoked by a sharp steel pin glued to the tip of a 1.0-g von Frey filament in *Nfia*^*F/F*^*;Wnt1-Cre* (*n* = 6) and *Nfia*^*F/F*^ control mice (*n* = 7) (Student’s unpaired *t-*test, *P < *0.0001, *t*_(11)_ = 14.63). **G** Noxious pinch-evoked licking responses in *Nfia*^*F/F*^ control and *Nfia*^*F/F*^*;Wnt1-Cre* mice (*n* = 7/group, Student’s unpaired *t-*test, *P* = 0.3472, *t*_(12)_ = 0.9783).

We next examined behavioral phenotypes in *Nfia*^*F/F*^*;Wnt1-Cre* mice, using littermates as blinded controls. We found that sensori-motor coordination was not affected in *Nfia*^*F/F*^*;Wnt1-Cre* mice (Fig. 4A), suggesting that NFIA is likely dispensable for proprioceptor development. We found that *Nfia*^*F/F*^ control mice and *Nfia*^*F/F*^*;Wnt1-Cre* mice showed comparable light touch responses using the dynamic brush assay (Fig. 4B). Reflexive responses to noxious heat or cold were also unaffected, as indicated by normal withdrawal latencies (Fig. 4C, D). We then found that the responses to light punctate pressure evoked by von Frey filaments were attenuated, with the withdrawal threshold increased from 0.29 ± 0.03 g in control mice to 0.46 ± 0.04 g in *Nfia*^*F/F*^*;Wnt1-Cre* mice (Fig. 4E). However, the net increase of average thresholds in CKO *versus* control mice (0.46 g – 0.29 g = 0.17 g) was modest compared with a much larger increase following the ablation of MrgprD^+^ neurons (0.99 g – 0.41 g = 0.58 g). This is consistent with a lack of change of MrgprD expression in *Nfia*^*F/F*^*;Wnt1-Cre* mice. As noted above, the Npy2r lineage neurons marked by transgenic *Npy2r-Cre* include the A-fiber nociceptors required to sense pinprick-evoked mechanical pain [8]. Consistent with a complete loss of Npy2r expression in *Nfia*^*F/F*^*;Wnt1-Cre* mice, pinprick-evoked responses were indeed markedly attenuated in CKO mice in comparison with control mice (Fig. 4F). In contrast, the licking responses evoked by skin-pinching stimuli, which produce sustained pain in humans [23], were unchanged (Fig. 4G).

**Fig. 5:**
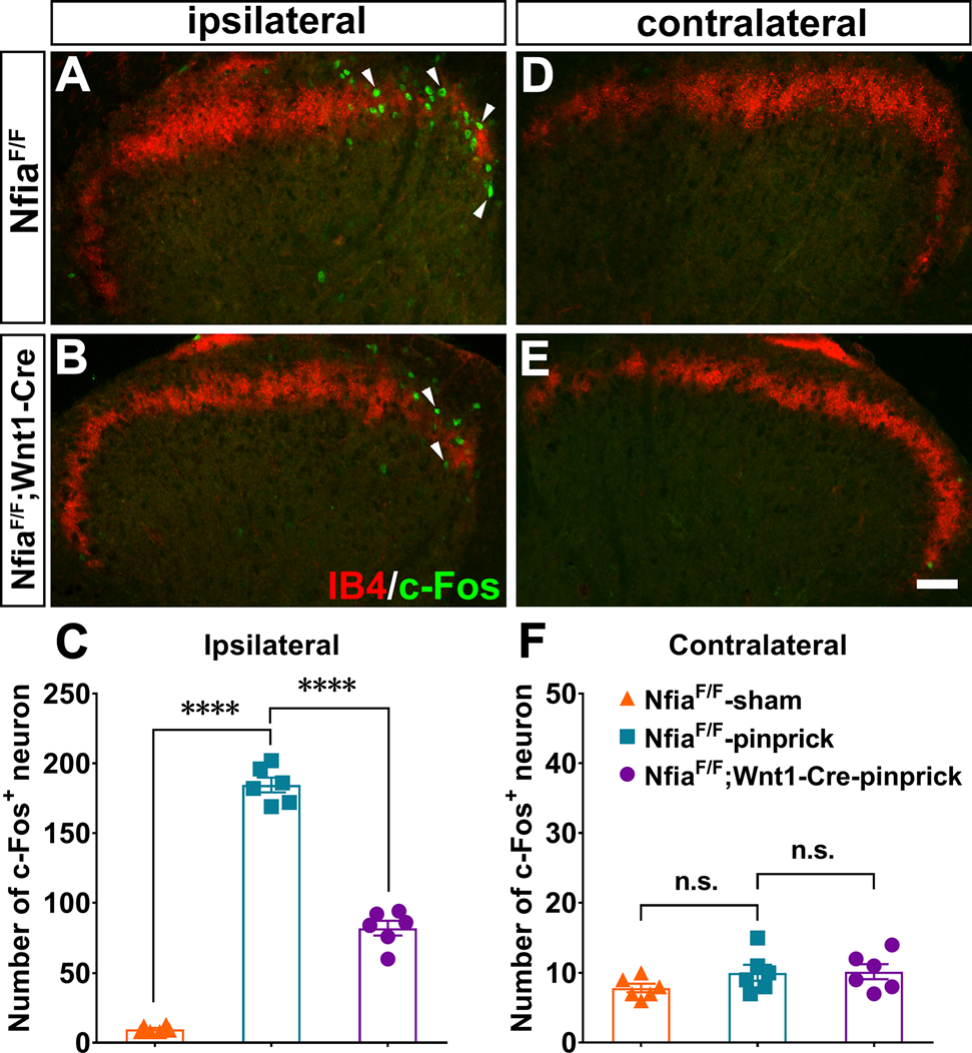
Impairment of pinprick-evoked information transmission in conditional *Nfia* null mice. **A, B, D, E** Representative images of pinprick-induced c-Fos expression in the ipsilateral and contralateral dorsal horn of adult control (**A, D**) and *Nfia*^*F/F*^*;Wnt1-Cre* mice (**B, E**) (scale bar, 100 µm). **C** Numbers of c-Fos^+^ neurons in the ipsilateral and contralateral dorsal horns from mice with indicated genotypes and conditions (*n* = 6/group, one-way ANOVA with Tukey’s *post-hoc* analysis, *F*_2, 15_ = 420.2, ****P < 0.0001). **F** Numbers of c-Fos^+^ neurons in the contralateral dorsal horns from mice with indicated genotypes and conditions (*n* = 6/group, one-way ANOVA with Tukey’s *post-hoc* analysis, *F*_2, 15_ =1.78, *P* = 0.2024).

To further confirm that the mutants had a deficit in transmitting pinprick-evoked sharp mechanical information, we examined the induction of c-Fos, an immediate-early protein induced by neural activity [30], in the dorsal spinal cord. To do this, a sharp needle glued to the tip of a 1.0-g von Frey filament was used to stimulate the left planta, and the c-Fos expression in the spinal segment innervated by L4 and L5 DRG neurons was assessed. We found a significant reduction in c-Fos expression in the ipsilateral dorsal horn from *Nfia*^*F/F*^*;Wnt1-Cre* mice (82.0 ± 5.1), in comparison with *Nfia*^*F/F*^ control mice (184.5 ± 5.3, *n* = 6/group, one-way ANOVA with Bonferroni *post-hoc* analysis, *F*_2, 15_ = 420.2, *P* < 0.0001; Fig. 5A–C). No significant c-Fos induction in the contralateral dorsal horn was observed in either *Nfia*^*F/F*^ control or *Nfia*^*F/F*^*;Wnt1-Cre* mice (*n* = 6/group, one-way ANOVA with Bonferroni *post-hoc* analysis, *F*_2, 15_ = 1.78, *P* = 0.2024; Fig. 5D–F). Thus, transmission of pinprick-evoked mechanical information from the plantar skin to the dorsal spinal cord is compromised in *Nfia*^*F/F*^*;Wnt1-Cre* mice.

**Fig. 6:**
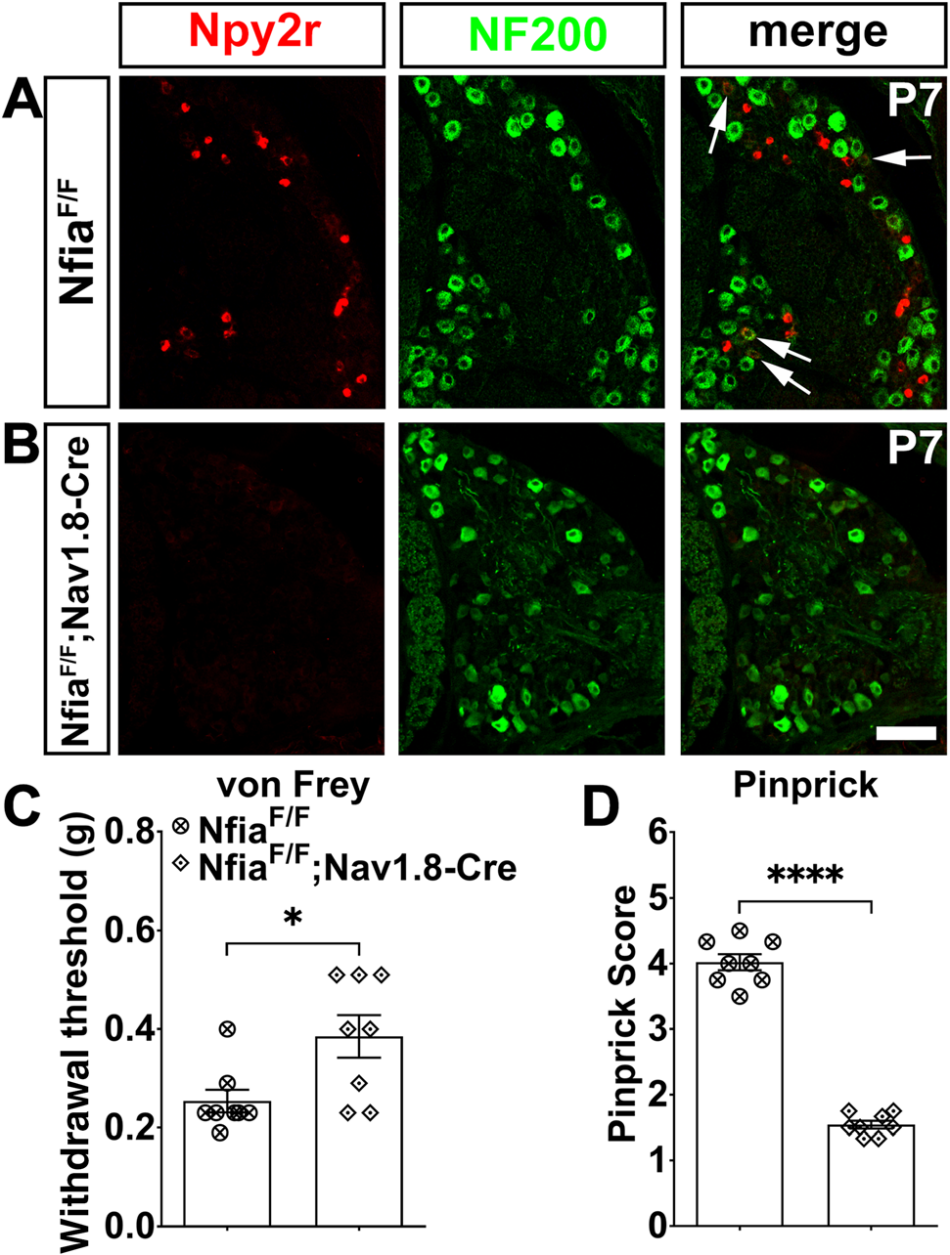
Requirement of NFIA for the induction of pinprick-evoked pain transmission**. A, B** Representative images of ISH with the Npy2r probe plus NF200 immunostaining in sections through lumbar DRGs from P7 *Nfia*^*F/F*^ control and *Nfia*^*F/F*^*;Nav1.8-Cre* mutant mice (arrows, Npy2r^+^;NF200^+^ neurons; scale bar, 100 µm). **C** Withdrawal thresholds in response to von Frey filament stimulation in *Nfia*^*F/F*^*;Nav1.8-Cre* CKO and *Nfia*^*F/F*^ control mice (*n* = 8/group, Student’s unpaired *t-*test, *P* = 0.0178, *t*_(14)_ = 2.683). **D** Pinprick scores evoked by a sharp steel pin glued to the tip of 1.0-g von Frey filament in *Nfia*^*F/F*^*;Nav1.8-Cre* and *Nfia*^*F/F*^ control mice (*n* = 8/group, Student’s unpaired *t-*test, *P* < 0.0001, *t*_(14)_ = 18.21).

It should be noted that in *Nfia*^*F/F*^*;Wnt1-Cre* mice, NFIA expression would have been eliminated in both DRGs and in the dorsal spinal cord [16]. However, the same loss of Npy2r expression and impairment of pinprick-evoked responses were found in *Nfia*^*F/F*^*;Nav1.8-Cre* mice (Fig. 6A–D), in which the Cre recombinase is confined to DRG neurons, not in spinal neurons [18, 31].

Thus, NFIA-dependent DRG neurons, which include the Npy2r lineage neurons, play a prominent role in the transmission of pinprick-evoked sharp mechanical stimuli. These neurons only play a minor role in the transmission of von Frey filament punctate pressure information and are dispensable for the processing of pinch-evoked sustained licking responses.

### Runx1 is Dispensable for the Development of Npy2r-transient A-fiber Neurons

**Fig. 7:**
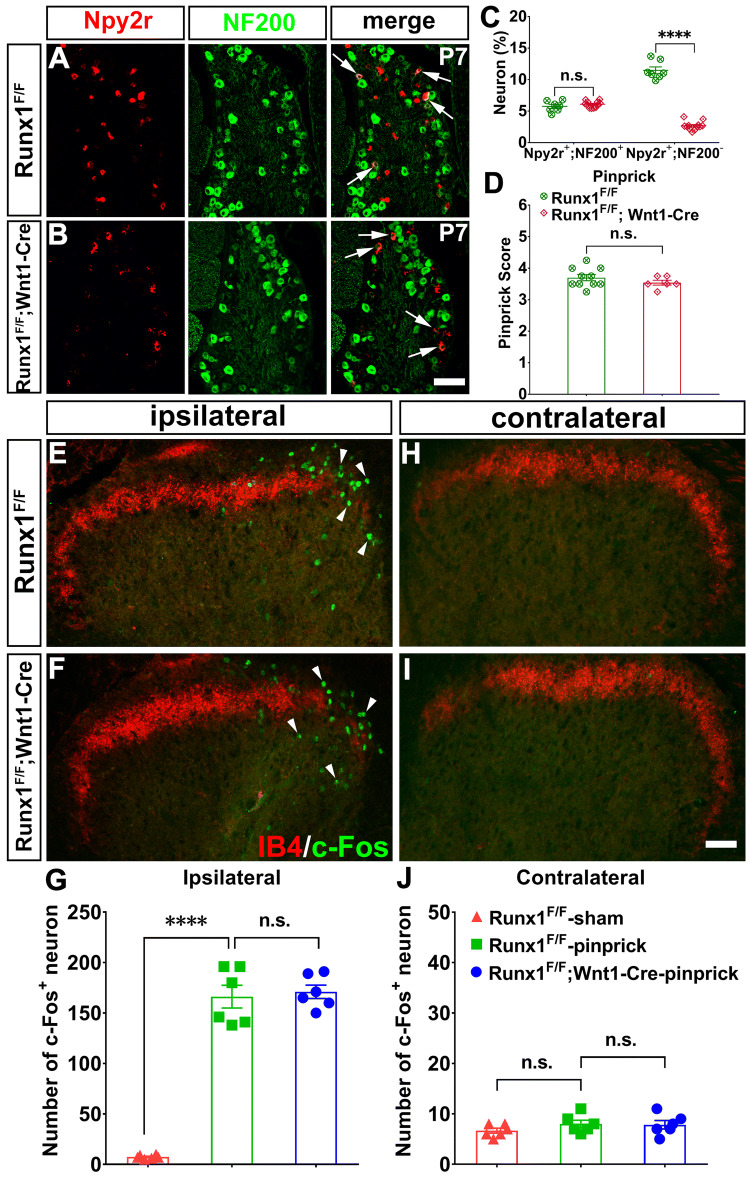
Requirement of Runx1 for the development of Npy2r-persistent but not Npy2r-transient neurons**. A, B** Representative images of ISH with the Npy2r probe plus NF200 immunostaining in sections through lumbar DRGs from P7 *Runx1*^*F/F*^ control and *Runx1*^*F/F*^*;Wnt1-Cre* mutant mice (arrows, Npy2r^+^;NF200^+^ neurons; scale bar, 100 µm). **C** Percentages of Npy2r^+^;NF200^+^ and Npy2r^+^;NF200^−^ among all neurons in *Runx1*^*F/F*^ control and *Runx1*^*F/F*^*;Wnt1-Cre* CKO mice. **D** Pinprick scores evoked by a sharp steel pin glued to the tip of 1.0 g von Frey filament in *Runx1*^*F/F*^ control (*n* = 10) and *Runx1*^*F/F*^*;Wnt1-Cre* mice (*n* = 6) (Student’s unpaired *t-*test, *P* = 0.2766, *t*_(14)_ = 1.132). **E, F** Pinprick-evoked c-Fos expression in the ipsilateral dorsal horns in *Runx1*^*F/F*^ control and *Runx1*^*F/F*^*;Wnt1-Cre* CKO mice. **G** Statistical comparison of c-Fos^+^ neuronal numbers in the ipsilateral dorsal horns between *Runx1*^*F/F*^ control and *Runx1*^*F/F*^*;Wnt1-Cre* CKO mice (*n* = 6/group, one-way ANOVA with Tukey’s *post-hoc* analysis, *F*_2, 15_ =151.7, ****P < 0.0001). **H, I** C-Fos in the contralateral dorsal horn in *Runx1*^*F/F*^ control and *Runx1*^*F/F*^*;Wnt1-Cre* CKO mice. **J** Statistical comparison of c-Fos^+^ neuronal numbers in the contralateral dorsal horn between *Runx1*^*F/F*^ control and *Runx1*^*F/F*^*;Wnt1-Cre* CKO mice (*n* = 6/group, one-way ANOVA with Tukey’s *post-hoc* analysis, *F*_2, 15_ =1.075, *P* = 0.366).

**Fig. 8:**
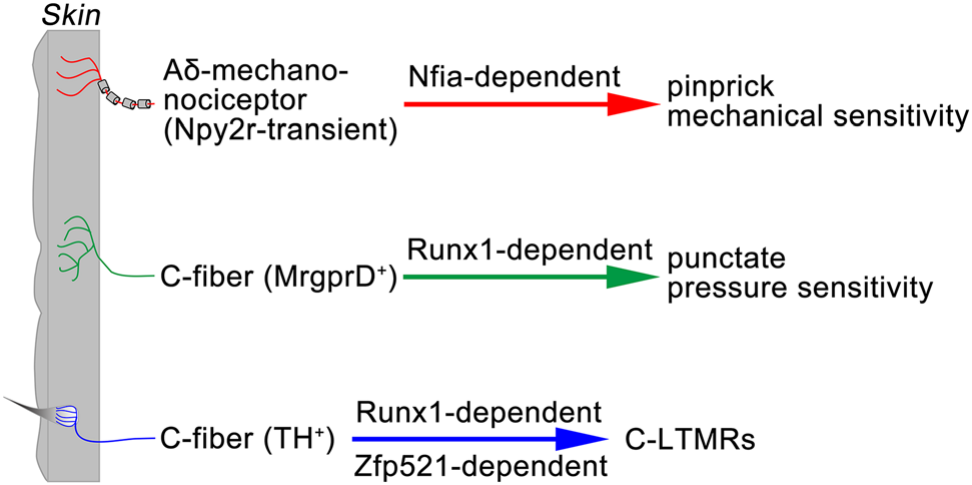
Separate genetic programs controlling different mechanoreceptors.

We have reported previously that the development of Npy2r-persistent (Nppb^+^) neurons requires the transcription factor Runx1 [24]. Hence we asked whether Runx1 is also involved in the development of Npy2r-transient myelinated sensory neurons. Using the same *Wnt1-Cre*, we generated *Runx1* CKO mice, referred to as *Runx1*^*F/F*^*;Wnt1-Cre*, and found that at P7, there was no difference between CKO and control mice in the percentages of myelinated neurons co-expressing Npy2r and NF200 (control: 5.8% ± 0.3%, *n* = 7; CKO: 6.1% ± 0.1%; *n* = 12; *t-*test, *P* = 0.8246, *t*_(34)_ = 0.83; Fig. 7A–C). In contrast, the percentages of Npy2r^+^;NF200^-^ neurons, which represent Npy2r-persistent unmyelinated Nppb^+^ pruriceptors (see above, Fig. 1J), were lower in P7 *Runx1*^*F/F*^*;Wnt1-Cre* mice than in control littermates (control: 11.5% ± 0.5%, *n* = 7; CKO: 2.7% ± 0.2%; *n* = 12; *t-*test, *P < *0.0001, *t*_(34)_ = 21.77; Fig. 7A–C), and we reported previously that by P30, persistent Npy2r expression is completely eliminated in these mutants [24]. Thus, Runx1 is required for the development of Npy2r-persistent unmyelinated pruriceptors, but is apparently dispensable for that of Npy2r-transient myelinated sensory neurons. We then found that pinprick-evoked responses remained unchanged in *Runx1*^*F/F*^*;Wnt1-Cre* mice compared with controls (control: 3.7 ± 0.1, *n* = 10; CKO: 3.5 ± 0.1; *n* = 6; *t-*test, *P* = 0.2766, *t*_(14)_ = 1.132; Fig. 7D). Furthermore, the pinprick-evoked c-Fos induction in the dorsal spinal cord was not affected in these mutants (control: 166.2 ± 11.3, *n* = 6; CKO: 171.0 ± 6.6; *n* = 6; one-way ANOVA with Bonferroni *post-hoc* analysis, *F*_2, 15_ = 151.7, *P* > 0.99; Fig. 7E–G), in contrast with the marked reduction in *Nfia*^*F/F*^*;Wnt1-Cre* mice. Our studies therefore suggested that Nfia and Runx1 control the development of different groups of mechanoreceptors (summarized in Fig. 8).

## Discussion

### NFIA Controls A-fiber Nociceptor Development

In humans, pinprick-evoked sharp pain percepts are mediated by myelinated A-fiber mechanoreceptors [32]. In both humans and animals, the transmission of noxious sensory information is dependent on TrkA-lineage neurons (TrkA is the receptor for nerve growth factor), since patients with loss-of-function *TrkA* mutations show congenital insensitivity to pain [33]. Earlier studies have shown that the genesis of TrkA neurons is entirely dependent on the proneural gene Neurog1 in the trigeminal and most rostral cervical DRGs [11]. Subsequent studies have shown that in thoracic and caudal DRGs, TrkA linages are formed in two waves, the early one giving rise to myelinated neurons and dependent on Neurog2, and the late one forming unmyelinated neurons and dependent on Neurog1 [9, 10], providing the first evidence for the existence of separate genetic programs controlling the development of myelinated *versus* unmyelinated nociceptors. Most recently, it has been reported that a subset of TrkA^+^ A-fiber nociceptors can be genetically marked by *Npy2r-Cre*, and ablation of these neurons leads to impaired responses to pinprick stimuli [8]. It should, however, be noted that *Npy2r-Cre*-marked neurons include those with persistent or transient *Npy2r* expression. Our studies suggested that Npy2r is expressed transiently in A-fiber sensory neurons, and persistent high level of Npy2r expression is confined to Nppb^+^ pruriceptors, consistent with single-cell RNA-seq results [25–27] and with an earlier report showing that Npy2r is predominantly expressed in small CGRP-positive DRG neurons [34]. Our subsequent studies showed that the transcription factor NFIA is required for the proper development of both Npy2r-transient and Npy2r-persistent neurons, as indicated by the complete elimination of Npy2r expression in *Nfia*^*F/F*^*;Wnt1-Cre* mice at both neonatal and young adult stages. Two potential mechanisms, peripheral sensitization and central disinhibition, are responsible for the development of pinprick-evoked pain [8]. The spinal neurons marked by *SOM*^*Cre*^ are critical for the transmission of both pinprick and von Frey hair stimuli [20, 35]. Conditional knock-out of *Nfia* confined to peripheral DRG neurons results in the same loss of Npy2r expression and impairment of pinprick-evoked responses, which indicates that NFIA is mainly responsible for the induction of pinprick-evoked mechanical pain. All together, the above findings suggest that NFIA-dependent Npy2r-transient DRG neurons represent A-fiber nociceptors that transmit noxious sharp mechanical information. In other words, our studies identify NFIA as the first post-mitotic transcription factor controlling the development of A-fiber nociceptors. The complete loss of Npy2r also suggests a requirement of NFIA for the proper development of Nppb^+^ neurons, which function as pruriceptors [28, 29]. Indeed, marked itch deficits were found in *Nfia*^*F/F*^*;Wnt1-Cre* mice (unpublished results to be described elsewhere).

### Separate Genetic Programs Controlling Myelinated *Versus* Unmyelinated Mechanical Nociceptors, as well as C-LTMRs

We found that while NFIA is necessary for the development of Npy2r-transient A-fiber nociceptors, it is dispensable for the expression of MrgprD, a marker for polymodal nociceptors, and TH, a marker for C-LTMRs, both of which are unmyelinated C-fiber sensory neurons [1–3, 5, 7]. Interestingly, the phenotypes are exactly opposite in mice with *Runx1* knockout, with complete loss of MrgprD and TH [6, 13] and without loss of Npy2r^+^;NF200^+^ myelinated sensory neurons, even though both transcription factors are required for the development of Npy2r-persistent Nppb^+^ pruriceptors. Earlier studies have shown that Runx1 expression is confined to the Neurog1-dependent late-born TrkA neurons, as indicated by complete loss of Runx1 in *Neurog1*^*-/-*^ mice [9, 13, 36]. This explains why Runx1 is dispensable for the development of Npy2r-transient A-fiber nociceptors, which are likely formed during the Neurog2-dependent first wave of neurogenesis [9]. It should be noted that another transcription factor, Zfp521, is expressed selectively in TH^+^ C-LTMRs, and it acts to promote and suppress molecular features associated with TH^+^ C-LTMRs and MrgprD^+^ nociceptors, respectively [15], leading to further segregation of Runx1-dependent neurons. All together, these studies reveal separate genetic programs controlling the emergence of functionally distinct mechanoreceptor subtypes among TrkA-lineage neurons (summarized in Fig. 8).
